# Real-World Evidence for Psychiatric Disorders from the German Disease Analyzer Database: A Narrative Review

**DOI:** 10.3390/brainsci16010115

**Published:** 2026-01-21

**Authors:** Karel Kostev, Marcel Konrad, Jens Bohlken

**Affiliations:** 1 Marburg University, University Hospital, 35043 Marburg, Germany; 2 Epidemiology, IQVIA, 60549 Frankfurt am Main, Germany; 3 Health & Social, FOM University of Applied Sciences for Economics and Management, 60486 Frankfurt am Main, Germany; 4 Faculty of Medicine, Institute of Social Medicine, Occupational Health and Public Health, University of Leipzig, 04109 Leipzig, Germany

**Keywords:** psychiatric disease, depression, bipolar disorder, somatic disease, COVID-19, cognitive disturbance, database

## Abstract

The German IQVIA Disease Analyzer (DA) database has become an increasingly important source of real-world evidence for psychiatric research. Over the past decade, and particularly since 2020, DA-based studies have addressed a broad spectrum of psychiatric outcomes including depression, anxiety disorders, schizophrenia, bipolar disorder, dementia, sleep disorders, and the mental health consequences of chronic somatic diseases and of contracting COVID-19. Using large, representative outpatient cohorts, these studies have examined factors associated with the incidence of psychiatric disorders, patterns of psychiatric and somatic comorbidity, treatment trajectories, and long-term outcomes under routine care conditions. The DA database’s longitudinal structure, nationwide coverage, and inclusion of multiple medical specialties enable it to capture psychiatric disorders throughout patient lifetimes and across different clinical contexts. This narrative review summarizes psychiatric research using the DA database that has been published since 2020, focusing on study design, main findings, methodological strengths and limitations, and implications for future psychiatric epidemiology and clinical research.

## 1. The Disease Analyzer Database as a Platform for Psychiatric Research

Psychiatric disorders are increasingly conceptualized as conditions embedded within complex, longitudinal health trajectories rather than as isolated disease entities. Large-scale epidemiological studies have demonstrated that mental disorders are frequently associated with a wide range of somatic and neurological conditions, including cardiovascular disease, metabolic disturbances, immune and inflammatory disorders, chronic pain, and neurodegenerative diseases such as dementia [1,2]. Depending on age, disease burden, and clinical context, psychiatric disorders may precede, follow, or co-occur with somatic and cognitive conditions, reflecting shared vulnerability factors, cumulative stress exposure, and sustained impairment in quality of life.

Conversely, chronic somatic illnesses, pain-related conditions, infectious diseases including COVID-19, and neurodegenerative disorders have been shown to increase the risk of subsequent psychiatric disturbances, likely mediated by chronic stress, reduced functional capacity, and allostatic load [3,4]. This bidirectional interplay provides a strong rationale for the use of large-scale electronic health record databases in psychiatric research, as such data allow the investigation of temporal associations, comorbidity structures, and treatment trajectories under real-world outpatient care conditions.

The IQVIA Disease Analyzer database consists of anonymized longitudinal data extracted directly from the electronic medical records of outpatient practices in Germany. Participating practices include general practitioners, psychiatrists, neurologists, pediatricians, gynecologists, and other specialists involved in routine patient care. The data recorded encompass ICD-10 diagnoses, ATC-coded prescriptions, consultation dates, patient age and sex, and selected clinical parameters such as body mass index or laboratory values, where available.

Psychiatric disorders are predominantly diagnosed and managed in outpatient settings, often over extended periods and in close interaction with somatic care. Consequently, the DA database is a particularly suitable source of data for psychiatric research. Unlike hospital-based datasets, which primarily capture acute episodes or severe disease, the DA database enables the study of early diagnostic phases, long-term follow-up, and treatment patterns in everyday clinical practice. This is especially relevant in the field of psychiatric epidemiology, where understanding disease burden requires knowledge of the timing of diagnosis, continuity of care, and chronicity of symptoms.

Another important feature of the DA is that it covers a wide range of medical specialties. Many psychiatric disorders are initially identified or managed outside of psychiatric practices, for example in general practice, pediatric care, or specialist somatic settings. The DA database facilitates the analysis of diagnostic pathways, referral patterns, and comorbidity constellations across these settings, reflecting the complexity of real-world psychiatric care.

The present narrative review focuses on psychiatric studies using the Disease Analyzer database that have been published since 2020. This temporal focus reflects several developments in the field. First, this period marks a substantial expansion and diversification of psychiatric research using the database, with an increasing number of large-scale, methodologically comparable cohort studies addressing long-term outcomes, cross-diagnostic associations, and treatment-related questions. Second, the COVID-19 pandemic led to the rapid emergence of new research priorities, including pandemic-related mental health effects and post-infectious neuropsychiatric outcomes, which could not have been covered in earlier summaries. Finally, compared with previous overviews of Disease Analyzer-based research that primarily introduced the database or reported selected findings, the present review provides a focused synthesis of recent psychiatric evidence with an emphasis on methodological patterns, interpretative coherence, and clinical relevance.

From a conceptual perspective, the breadth of psychiatric outcomes captured in the Disease Analyzer database aligns closely with contemporary approaches in psychiatric epidemiology that emphasize life-course trajectories, multimorbidity, and systems-based models of mental health. Within these frameworks, psychiatric disorders are understood not as isolated events, but as dynamic components of longitudinal health trajectories shaped by cumulative somatic disease burden, healthcare utilization, and broader contextual factors. The ability of routine outpatient data to capture psychiatric diagnoses alongside chronic somatic conditions across extended periods makes the Disease Analyzer database particularly well suited for examining mental health within such integrative epidemiological models.

Methodologically speaking, most DA-based psychiatric studies employ retrospective cohort or nested case–control designs. Matching procedures and multivariable regression models are frequently used to account for differences in age, sex, consultation frequency, and comorbidities. Follow-up periods range from one year in pandemic-related analyses to ten years in studies of long-term outcomes, such as dementia or cancer diagnoses. Table 1 provides an overview of Disease Analyzer-based psychiatric studies published since 2020, including study design, setting, sample size, and follow-up duration.

The present review is narrative in nature. Relevant studies were identified through targeted searches of the scientific literature focusing on psychiatric research conducted using the German IQVIA Disease Analyzer database and published since 2020. The aim was to capture the breadth of recent Disease Analyzer-based psychiatric research rather than to perform a formal systematic review. Studies were included if they examined psychiatric disorders, cognitive outcomes, or psychopharmacological aspects using Disease Analyzer data; no additional restrictions regarding study design or outcome were applied. While we sought to include all major publications in this area during the specified period, the review does not claim formal exhaustiveness.

As shown in Table 1, the studies published since 2020 encompass a wide range of psychiatric conditions, with a particular emphasis on mood and anxiety disorders, severe mental illness, dementia, and psychopharmacological treatment patterns. Across these works, associations between psychiatric disorders and chronic somatic disease, healthcare utilization, and long-term outcomes are reported more frequently than rare or highly specific psychiatric conditions, reflecting both disease prevalence in routine care and the strengths of large outpatient databases for studying common and clinically relevant conditions.

In the following sections, we describe these studies in more detail with respect to the methodologies adopted and the associations reported. We first outline common methodological approaches used in Disease Analyzer-based psychiatric research and then discuss individual disease areas and associations separately, following the structure of Table 1

## 2. Methodological Approaches in Disease Analyzer-Based Psychiatric Studies

Most psychiatric studies using the IQVIA Disease Analyzer database apply a limited number of recurring methodological frameworks tailored to routine outpatient data. The most common approach is the retrospective cohort design, in which patients with a defined index diagnosis or exposure are followed longitudinally and compared with matched control groups who do not have the condition of interest. Matching is commonly performed based on age, sex, index year, and consultation frequency, reflecting the importance of healthcare utilization as a potential source of bias in psychiatric research.

In studies examining psychiatric outcomes following somatic disease, cohort entry is typically defined as the first documented diagnosis of the somatic condition. Patients with prior psychiatric diagnoses are excluded to ensure temporal ordering. Follow-up continues until the first psychiatric diagnosis, loss to follow-up, or end of data availability. This design has been applied consistently across studies investigating depression and anxiety disorders following chronic kidney disease, inflammatory bowel disease, dermatological conditions, and other somatic illnesses.

Nested case–control designs are used less frequently, but have been employed in pharmacoepidemiological research, particularly in studies examining dementia outcomes associated with long-term medication exposure. In these analyses, patients with incident dementia are matched to controls based on age, sex, and observation time. This allows exposure histories to be evaluated efficiently within large source populations.

The methodological approaches described below are derived from and exemplified by the studies summarized in Table 1, which form the empirical basis of this narrative review.

Outcome ascertainment in DA-based psychiatry studies relies on ICD-10 diagnostic codes documented in routine care. To increase diagnostic specificity, many studies require a diagnosis to be documented repeatedly or confirmed in specialist care. Prescription of disorder-specific medications is sometimes used as an additional indicator of clinically relevant disease, particularly for depression and dementia.

Multivariable regression models are commonly employed across studies to adjust for comorbid somatic conditions and baseline medication use. While residual confounding cannot be entirely ruled out, consistent methodological approaches across different disease areas facilitate the comparison of findings and support the internal coherence of the DA-based psychiatric literature.

## 3. Mood and Anxiety Disorders

### 3.1. Depression and Anxiety Following Somatic Disease

The occurrence of depression and anxiety disorders following chronic somatic disease diagnoses has been a major focus of DA-based psychiatric research. Multiple large retrospective cohort studies consistently demonstrate higher incidences of depression and anxiety disorders among patients with chronic medical conditions diagnosed and treated in routine outpatient care.

Such associations have been reported for chronic kidney disease [17], inflammatory bowel disease [26], psoriasis [30], hemorrhoids [19], carpal tunnel syndrome [15], and gynecological conditions managed in specialist practices [28]. These studies typically employed matched cohort designs and demonstrated that the associations observed persisted over several years of follow-up rather than being limited to the period immediately following diagnosis.

The COVID-19 pandemic has also been examined as a potential risk factor for incident mood and anxiety disorders. In a large retrospective cohort study conducted in German primary care, no significant association was observed between documented COVID-19 diagnosis and the subsequent incidence of depression or anxiety disorders compared with matched controls [14]. These findings suggest that, at a population level, SARS-CoV-2 infection alone may not have been a dominant driver of new affective or anxiety disorder diagnoses in routine outpatient care.

Complementing these findings, a cross-sectional DA-based study assessed the overall burden of stress-related, anxiety, and depressive disorders during the COVID-19 pandemic in German primary care. The study reported increased documentation of stress-related and affective disorders during pandemic periods compared with pre-pandemic baselines, highlighting the broader mental health impact of societal disruption and healthcare system strain rather than infection status alone [7]. These observations are consistent with international evidence indicating that the mental health impact of the COVID-19 pandemic at the population level was largely driven by societal stressors, healthcare disruption, and psychosocial burden rather than infection alone, as summarized in recent scoping reviews of pandemic-related psychiatric outcomes, including suicide-related behavior [31].

From a clinical perspective, these findings highlight the close interrelationship between chronic physical illness and mental health. Psychiatric morbidity may emerge in the context of persistent symptoms, pain, functional impairment, uncertainty regarding prognosis, or repeated contact with healthcare professionals. While DA-based analyses cannot address psychosocial stressors or inflammatory mechanisms directly, they provide robust population-level evidence that mood and anxiety disorders are frequently embedded within long-term somatic disease trajectories encountered in everyday practice.

### 3.2. Depression as an Exposure

In addition to examining depression as an outcome, several DA-based studies have investigated depression as an exposure factor associated with subsequent non-psychiatric diagnoses. Large matched cohort analyses have reported an association between depression and higher incidences of subsequent cancer [23] and dementia [27] diagnoses.

These associations may reflect shared biological pathways, behavioral factors, or differences in healthcare utilization and diagnostic intensity. Depression is often accompanied by increased medical contact, which may contribute to the earlier detection of somatic disease. Conversely, prodromal somatic illness may initially manifest as depressive symptoms. However, because the DA database lacks detailed information on lifestyle factors, socioeconomic status, and subclinical disease at baseline, causal interpretations remain limited. Nevertheless, these findings underline the importance of considering depression within the broader context of general health vulnerability in outpatient populations.

## 4. Severe Mental Illness: Schizophrenia and Bipolar Disorder

### 4.1. Schizophrenia and Somatic Disease Patterns

Several DA-based studies have addressed severe mental illnesses, with a particular emphasis on schizophrenia. A large retrospective cohort analysis demonstrated that schizophrenia was associated with a distinct cardiovascular disease profile, including higher rates of heart failure but lower documented rates of atrial fibrillation and several other cardiovascular diagnoses [25]. This pattern may reflect a combination of biological factors, metabolic effects of antipsychotic medication, lifestyle differences, and the underdiagnosis of somatic diseases in patients with severe mental illness.

Another DA-based cohort study reported a negative association between schizophrenia and subsequent cancer diagnoses [24]. Although counterintuitive, this finding aligns with observations from other real-world datasets and raises important questions regarding competing mortality, reduced participation in cancer screening programs, and diagnostic overshadowing in clinical practice. Together, these studies illustrate how DA data can be used to investigate health disparities and diagnostic patterns affecting patients with severe mental illness.

### 4.2. Bipolar Disorder and Antidepressant-Associated Outcomes

Bipolar disorder has primarily been examined from a treatment-related perspective. A large real-world study investigated factors associated with switches to mania or hypomania in patients with bipolar depression who were being treated with antidepressants [12]. The analysis revealed differences between antidepressant classes, reflecting variations in routine prescribing practices and patient responses in everyday clinical settings.

In addition to studies on antidepressant-associated switching, DA-based research has examined real-world treatment outcomes in bipolar disorder under routine care conditions. A retrospective cohort study compared outcomes of monotherapy with lithium, valproate, quetiapine, olanzapine, venlafaxine, and citalopram in patients with bipolar disorder treated in German outpatient practices. The analysis demonstrated marked differences in clinical outcomes between mood stabilizers, antipsychotics, and antidepressants, reflecting heterogeneity in real-world prescribing and effectiveness beyond controlled trial settings [6]. These findings underscore the value of routine care data for evaluating comparative treatment outcomes in bipolar disorder, particularly in patient populations often underrepresented in randomized clinical trials.

The observed differences in clinical outcomes between pharmacological treatment classes in bipolar disorder are consistent with their distinct pharmacodynamic profiles. Mood stabilizers such as lithium and valproate exert effects on intracellular signaling pathways, synaptic plasticity, and neuroprotective mechanisms, which are thought to contribute to relapse prevention and long-term mood stabilization [32]. Second-generation antipsychotics, including quetiapine and olanzapine, combine dopaminergic and serotonergic receptor modulation and are widely used for both acute mood episodes and maintenance treatment [33]. In contrast, antidepressants lack intrinsic mood-stabilizing properties and may increase the risk of affective switching in susceptible patients, a phenomenon consistently reported in both clinical trials and real-world studies [34,35]. While causal inference is not possible in observational routine care data, these real-world differences plausibly reflect underlying pharmacological mechanisms expressed under everyday clinical conditions.

Such studies complement randomized controlled trials by incorporating broader patient populations, including older adults and individuals with multiple comorbidities who are often excluded from trials. At the same time, confounding by indication and treatment selection remains an important limitation of observational pharmacoepidemiological research, underscoring the need for cautious interpretation of findings.

## 5. Sleep Disorders and Psychiatric Outcomes in Children and Adolescents

Children and adolescents constitute an important subgroup in DA-based psychiatric research. A large retrospective cohort study demonstrated that sleep disorders were associated with higher incidences of subsequent depression and anxiety disorders in this age group (Kostev et al., 2025 [20]). Sleep complaints are frequently encountered in pediatric practice and may represent early manifestations of subsequent psychiatric disorders.

The COVID-19 pandemic provided a unique context for temporal analyses. Several DA-based studies have documented marked increases in depression and anxiety disorder diagnoses among children and adolescents during the pandemic compared to pre-pandemic periods [18,29]. These analyses benefit from large sample sizes and consistent data collection before and during the pandemic, facilitating robust comparisons within the same healthcare system.

Furthermore, prescription-focused studies have demonstrated age-specific patterns of antidepressant use among adolescents newly diagnosed with depression, highlighting variation in pharmacological management across different practices and regions [16]. Together, these findings illustrate how DA data can capture both diagnostic and treatment-related aspects of child and adolescent mental health.

## 6. Dementia and Cognitive Disorders

### 6.1. Factors Associated with Incident Dementia

Long-term trends in dementia and mild cognitive impairment diagnoses have been systematically evaluated using DA data. An analysis of German general and specialist practices between 2015 and 2019 demonstrated stable to moderately increasing prevalence and incidence rates of dementia and mild cognitive impairment, with variations by care setting [8]. In routine outpatient data, mild cognitive impairment is documented using ICD-10 codes related to mild cognitive disorders; for clarity, the term mild cognitive impairment (MCI) is used throughout this review unless referring explicitly to coding practices. These findings provide important baseline information for interpreting subsequent pandemic-related and post-COVID cognitive outcomes within routine outpatient care.

Beyond incidence trends, DA-based research has examined the documentation of prodromal features and risk factors preceding dementia diagnoses. A recent study showed that cognitive symptoms, vascular risk factors, psychiatric comorbidities, and functional complaints were frequently documented in primary care prior to formal dementia diagnosis, although substantial heterogeneity in documentation practices was observed [21]. These results highlight both the potential and the limitations of routine care data for early dementia detection and risk stratification in primary care settings.

Dementia is one of the most extensively studied outcomes in DA-based psychiatric research. Several large cohort studies have examined the associations between somatic diseases such as psoriasis [30], cancer [26], and COVID-19 infection [13] and subsequent dementia diagnoses. These analyses consistently demonstrate that dementia diagnoses are closely intertwined with overall somatic disease burden and healthcare utilization in older adults.

Post-infectious cognitive outcomes have received particular attention. Analyses of primary care patients with a history of documented COVID-19 infection have revealed higher incidences of mild cognitive disorder and dementia compared to uninfected controls or those with other respiratory infections [9,13]. These findings suggest that the cognitive sequelae of COVID-19 can be detected in routine outpatient data and may persist beyond the acute phase of infection, even when severe acute illness has not been documented.

### 6.2. Dementia Treatment Patterns and Progression

DA data have also been used to explore treatment-related aspects of dementia. Two longitudinal cohort studies have reported an association between *Ginkgo biloba* extract prescriptions and lower dementia incidence as well as slower progression of dementia severity in patients with mild cognitive impairment [10,11]. While causal inference is limited by the observational design, these analyses illustrate how routine care data can be used to examine commonly prescribed therapies that are used widely in clinical practice, yet have been studied incompletely in randomized trials.

Additionally, a case–control study examined long-term antidepressant use and subsequent dementia diagnoses, revealing heterogeneous associations across drug classes and individual agents [27]. These findings highlight the complexity of pharmacoepidemiological research in elderly psychiatric populations and the importance of carefully interpreting observational evidence.

## 7. Cross-Diagnostic Synthesis of Psychiatric Findings from Disease Analyzer Studies

Several recurring patterns have emerged from psychiatric research using the DA database, across diagnostic categories. One of the most consistent observations is the close temporal association between chronic somatic disease and subsequent psychiatric diagnoses, particularly depression and anxiety disorders. This pattern has been documented in relation to a wide range of somatic conditions including chronic kidney disease [17], inflammatory bowel disease [26], dermatological diseases such as psoriasis [30], and functional or pain-related disorders [15,19]. The recurrence of this association across diverse disease groups suggests that psychiatric morbidity in routine care often reflects cumulative disease burden rather than organ-specific mechanisms alone.

Another cross-diagnostic theme concerns the role of healthcare contact in detecting psychiatric disorders. Many DA-based studies adjust for consultation frequency, acknowledging that increased medical contact may facilitate the identification and documentation of psychiatric symptoms. Nevertheless, associations between somatic disease and psychiatric outcomes typically persist after such adjustments have been made [17,26]. This suggests that differences in healthcare utilization alone cannot fully explain the findings observed.

In the context of severe mental illness, DA-based analyses highlight systematic discrepancies in the documentation of somatic disease. Studies of patients with schizophrenia demonstrate heterogeneous associations with cardiovascular diagnoses and a lower recorded incidence of certain cancers [24,25]. These patterns are consistent with concerns about underdiagnosis, diagnostic overshadowing, and disparities in preventive care for patients with severe mental illness. They also illustrate how routine outpatient data can be used to identify such phenomena at a population level.

Research on dementia and cognitive disorders further emphasizes the embeddedness of psychiatric outcomes within general medical care. Dementia diagnoses in the DA database are frequently observed following major somatic conditions or infectious events, including COVID-19 [9,13]. The consistent documentation of post-COVID cognitive disorders in primary care settings suggests that these syndromes are clinically apparent in routine practice and are not restricted to specialized memory clinics.

Finally, DA-based studies in children and adolescents highlight the importance of early-life circumstances and external stressors in shaping psychiatric trajectories. For example, sleep disorders in childhood have been associated with subsequent depression and anxiety diagnoses [20], while the COVID-19 pandemic has been linked to marked increases in affective and anxiety disorders in pediatric populations [18,29]. Together, these findings demonstrate that the DA database can capture psychiatric outcomes across patient lifetimes and under changing societal conditions.

Taken together, these cross-diagnostic patterns are consistent with life-course and multimorbidity frameworks in psychiatric epidemiology, in which mental disorders emerge through the accumulation and interaction of somatic illness, functional impairment, and sustained healthcare engagement over time. From a systems-based perspective, the findings reviewed here suggest that psychiatric diagnoses documented in routine care reflect not only disease-specific processes, but also the structure and dynamics of healthcare delivery itself. Making this perspective explicit helps to interpret Disease Analyzer-based findings as manifestations of longitudinal health system interactions rather than as isolated disease associations.

## 8. Psychopharmacology and Treatment Patterns

Beyond disease associations, Disease Analyzer-based studies provide important insights into psychopharmacological treatment patterns and their real-world outcomes. In bipolar disorder, comparative analyses of monotherapy demonstrate heterogeneous outcomes across mood stabilizers, antipsychotics, and antidepressants, reflecting both pharmacological differences and treatment selection in routine care. Mood stabilizers such as lithium and valproate are associated with more stable long-term outcomes, consistent with their established efficacy in relapse prevention [32], whereas antidepressants show more variable effectiveness and are linked to an increased risk of affective switching in some patient groups [34].

In schizophrenia, real-world prescribing patterns are strongly influenced by the metabolic and cardiovascular side-effect profiles of antipsychotic agents. Dopamine D2 receptor blockade, combined with varying affinities for histaminergic, muscarinic, and serotonergic receptors, contributes to weight gain, dyslipidemia, and insulin resistance [36]. These pharmacological effects are clinically relevant when interpreting DA-based findings on cardiovascular disease patterns in patients with schizophrenia.

In unipolar depression, age-specific differences in antidepressant prescribing reflect pharmacokinetic considerations, tolerability profiles, and safety concerns across the lifespan. Selective serotonin reuptake inhibitors are most frequently prescribed, in line with their favorable benefit–risk ratio compared with older antidepressant classes [37]. Observed heterogeneity in long-term outcomes in routine care likely reflects differences in adherence, comorbidity, and baseline severity rather than drug effects alone.

In the field of dementia, DA-based pharmacoepidemiological studies examining cholinergic agents, antidepressants, and *Ginkgo biloba* extract illustrate how commonly prescribed therapies can be evaluated in large outpatient populations. While causal inference remains limited, observational evidence suggests that certain interventions may be associated with differences in dementia incidence or progression, complementing findings from randomized controlled trials [38,39].

Overall, these findings show that Disease Analyzer data capture clinically meaningful differences in psychiatric treatment outcomes, while reflecting the complexity of real-world prescribing and patient heterogeneity.

## 9. Strengths and Limitations of the Disease Analyzer Database in Psychiatric Research

DA-based psychiatric research benefits from large sample sizes, nationwide coverage, and longitudinal follow-up in routine outpatient care. These characteristics allow researchers to study relatively rare outcomes, long-term associations between somatic and psychiatric disorders, and treatment trajectories across diverse patient groups.

At the same time, several limitations must be considered. Psychiatric diagnoses are based on ICD-10 codes without standardized information on symptom severity, functional impairment, or psychosocial context. Diagnostic practices may vary between physicians, leading to potential misclassification. Information on lifestyle factors, socioeconomic status, and psychotherapy content is not available directly, which may result in residual confounding.

A central methodological challenge in Disease Analyzer-based psychiatric research is the potential for diagnostic misclassification and variability in ICD-10 coding practices across medical specialties. Psychiatric diagnoses documented in general practice, pediatric care, or somatic specialist settings may differ in diagnostic certainty, threshold, and follow-up intensity compared with those recorded in psychiatric practices. Several studies included in this review attempted to mitigate this limitation by requiring repeated diagnoses or confirmation in specialist care; nevertheless, residual heterogeneity in diagnostic practices cannot be excluded and should be considered when interpreting incidence estimates and cross-setting comparisons.

Surveillance bias is a potential concern in observational studies linking somatic disease to subsequent psychiatric diagnoses; however, this issue is systematically addressed in Disease Analyzer-based research by matching patients on consultation frequency or by adjusting for visit frequency in multivariable models. This methodological approach substantially reduces bias arising from differential healthcare utilization between exposed and unexposed groups. Nevertheless, even after such adjustment, subtle differences in diagnostic attention or symptom disclosure across clinical contexts cannot be fully excluded in routine care data and should be considered when interpreting elevated psychiatric incidence following chronic somatic disease.

Confounding by indication is especially relevant in pharmacoepidemiological analyses, including studies of bipolar disorder treatments and dementia-related medication exposure. In bipolar disorder, treatment selection is closely linked to disease severity, polarity, and prior treatment response, which complicates the interpretation of outcome differences between mood stabilizers, antipsychotics, and antidepressants in routine care. Similarly, in dementia research, associations between specific pharmacological treatments and disease progression may reflect underlying differences in patient frailty, comorbidity, or stage of cognitive impairment rather than treatment effects per se. While matching procedures and multivariable adjustment reduce this bias, they cannot fully eliminate it in observational routine data.

Importantly, the consistency of findings across multiple Disease Analyzer-based studies using similar designs and across different disease contexts supports their internal coherence. Nevertheless, the examples discussed above underscore that observed associations should be interpreted as real-world patterns rather than causal effects, highlighting the need for complementary evidence from prospective and experimental research designs.

An additional consideration concerns the generalizability of findings derived from the German outpatient healthcare setting. Certain aspects of Disease Analyzer-based results are likely influenced by healthcare system-specific features, including universal health insurance coverage, relatively unrestricted access to specialist care, and national reimbursement and coding practices. These factors may shape diagnostic pathways, consultation patterns, and treatment choices in ways that are not directly transferable to healthcare systems with different organizational structures.

At the same time, many of the patterns observed across the reviewed studies—such as longitudinal associations between chronic somatic disease and subsequent psychiatric diagnoses, heterogeneity in psychopharmacological treatment outcomes, and the embedding of psychiatric disorders within long-term healthcare trajectories—are consistent with findings from other high-income countries using large electronic medical record or claims databases. As such, while absolute incidence estimates and care pathways may be context-dependent, the overarching epidemiological and conceptual insights are likely transferable to settings with comparable data infrastructures and healthcare utilization patterns.

Inpatient psychiatric care and hospital-only events are incompletely documented. Consequently, DA-based findings should be considered as complementary to evidence from clinical trials, registries, and prospective cohort studies, rather than as substitutes for them.

## 10. Future Directions

Psychiatric research based on the Disease Analyzer database offers several important perspectives for future research and clinical development. Across diagnostic categories, the reviewed studies consistently demonstrate that psychiatric disorders documented in routine outpatient care are closely intertwined with chronic somatic disease, healthcare utilization patterns, and long-term treatment trajectories. As summarized in Table 2, these recurring associations highlight opportunities for hypothesis generation and for prioritizing future research questions.

First, the repeated observation of elevated psychiatric incidence following specific somatic conditions suggests that routine outpatient data may help identify patient groups at increased mental health risk. While Disease Analyzer-based studies cannot directly evaluate screening interventions, they can inform hypotheses regarding optimal timing, clinical settings, and target populations for future prospective studies on early detection and preventive strategies in primary and specialist care.

Second, the close temporal relationship between somatic and psychiatric diagnoses observed across multiple disease contexts supports further investigation of integrated somatic–psychiatric care pathways. Real-world evidence documenting frequent diagnostic overlap and sequential disease trajectories may serve as a foundation for developing and evaluating collaborative care models that bridge medical specialties and address mental health needs within chronic disease management.

Third, pharmacoepidemiological findings derived from the Disease Analyzer database can guide hypothesis generation for prospective and interventional research. Observed heterogeneity in treatment outcomes—such as differences between pharmacological classes in bipolar disorder or associations between medication exposure and cognitive outcomes in dementia—can help prioritize research questions, identify clinically relevant subgroups, and inform the design of randomized, pragmatic, or comparative effectiveness studies.

Finally, future methodological advances may further enhance the translational value of Disease Analyzer-based psychiatric research. Linkage with hospital, mortality, or registry data could allow more comprehensive outcome assessment, while the application of causal inference approaches may improve the interpretability of complex longitudinal associations. Together, these directions underscore the potential of large-scale outpatient data not only to describe psychiatric epidemiology, but also to inform the next generation of hypothesis-driven and clinically oriented mental health research.

## 11. Conclusions

Since 2020, psychiatric research using the German IQVIA Disease Analyzer database has expanded substantially to encompass mood and anxiety disorders, severe mental illness, dementia, sleep disorders, and psychopharmacology. Collectively, these studies demonstrate that routine outpatient data can contribute meaningfully to the understanding of psychiatric disease burden, comorbidity patterns, and treatment trajectories in large populations.

Across diagnostic categories, the evidence summarized in this review consistently indicates that psychiatric disorders documented in routine care are closely intertwined with long-term somatic disease, cognitive outcomes, and sustained healthcare engagement. This perspective aligns with contemporary life-course and multimorbidity models in psychiatric epidemiology, in which mental disorders are conceptualized as integral components of longitudinal health trajectories rather than isolated disease entities.

Although Disease Analyzer-based research does not include direct biological measurements, the large-scale and longitudinal characterization of psychiatric, somatic, and cognitive trajectories in real-world outpatient settings can also support biological psychiatry by informing hypothesis generation, identifying clinically meaningful phenotypes, and guiding the design of mechanistic and biomarker-driven studies. Despite the inherent limitations of observational routine data, Disease Analyzer-based research therefore represents an important and complementary component of contemporary psychiatric epidemiology.

## Figures and Tables

**Table 1:** Overview of key Disease Analyzer studies in psychiatry.

First Author (Year) [Reference]	Psychiatric Outcome	Exposure/Main Question	Study Design	N (Patients)	Setting	Max. Follow-Up (Years)
Bohlken (2020) [5]	Compliance of patients	Proportion of days covered (PDC) per patient depending on physician	Cross-sectional	>5000	Neuropsychiatric	3
Bohlken (2021) [6]	Bipolar disorder outcomes	Monotherapy with mood stabilizers, antipsychotics, antidepressants	Retrospective cohort	>20,000	Neuropsychiatric/GP	2
Bohlken (2021) [7]	Stress, anxiety, depression	COVID-19 pandemic period	Cross-sectional	>1 M visits	GP	1
Bohlken (2021) [8]	Dementia, MCI	Time trends 2015–2019	Retrospective cohort	>2 M	GP/Neuropsychiatric	5
Bohlken (2022) [9]	Mild cognitive disorder	Post-COVID syndrome	Retrospective cohort	>67,000	GP	1
Bohlken (2022) [10]	Dementia incidence	*Ginkgo biloba* in MCI	Retrospective cohort	>24,000	GP	5
Bohlken (2024) [11]	Dementia severity progression	*Ginkgo biloba* extract	Longitudinal cohort	>6000	GP/Neuropsychiatric	5
Feng (2025) [12]	Switch to mania/hypomania	Antidepressants in bipolar depression	Retrospective cohort	>20,000	Neuropsychiatric	2
Gollop (2023) [13]	Incident dementia	COVID-19 vs. URI	Retrospective cohort	>60,000	GP	2
Jacob (2022) [14]	Depression, anxiety	COVID-19 diagnosis	Retrospective cohort	>150,000	GP	1
Jacob (2023) [15]	Depression and anxiety	Carpal tunnel syndrome	Retrospective cohort	>95,000	GP	5
Kaur (2024) [16]	Antidepressant prescribing	New adolescent depression	Descriptive cohort	>9000	Pediatric	1
Kommer (2025) [17]	Incident depression	Chronic kidney disease	Retrospective cohort	>82,000	GP	10
Kostev (2023) [18]	Depression and anxiety (youth)	COVID-19 pandemic	Retrospective cohort	>160,000	Pediatric	2
Kostev (2024) [19]	Incident depression	Hemorrhoids	Retrospective cohort	>160,000	GP	5
Kostev (2025) [20]	Depression and anxiety (youth)	Sleep disorders	Retrospective cohort	>70,000	Pediatric	5
Michalowsky (2024) [21]	Dementia	Prodromal features, risk factors	Retrospective cohort	>40,000	GP	5
Mössinger (2021) [22]	Antidepressant treatment patterns	Depression	Retrospective cohort	>138,000	GP	1
Mössinger (2023) [23]	Cancer incidence	Depression	Retrospective cohort	>235,000	GP	10
Rodemer (2024) [24]	Cancer incidence	Schizophrenia	Retrospective cohort	>52,000	GP	10
Rodemer (2025) [25]	Cardiovascular diseases in schizophrenia	Schizophrenia vs. controls	Retrospective cohort	>75,000	GP	10
Roderburg (2024) [26]	Depression and anxiety	Inflammatory bowel disease	Retrospective cohort	>31,000	GP	5
Bartels (2020) [27]	Dementia	Antidepressant therapy	Case–control	>124,000	GP	5
Drewes (2021) [28]	Depression	Women in gynecologist practices	Case–control	>11,000	Gynecologists	1
Weber (2022) [29]	Psychiatric diagnoses	COVID-19 pandemic	Descriptive cohort	>1 M visits	Pediatric	2
Zingel (2023) [30]	Dementia	Psoriasis	Retrospective cohort	>70,000	GP	10

**Table 2:** Main psychiatric outcomes, observed associations, and implications for future research and care. Associations are based on observational routine outpatient data and should not be interpreted as causal.

Exposure–Outcome Constellation	Outcome Category	Typical Effect Direction	Clinical Interpretation	Implications for Future Research and Care
Chronic somatic disease → depression/anxiety	Mood and anxiety disorders	Higher incidence	Psychiatric morbidity frequently accompanies chronic illness	Identification of high-risk populations for targeted screening and evaluation of integrated somatic–psychiatric care models
Sleep disorders → depression/anxiety (youth)	Child & adolescent psychiatry	Strong positive association	Sleep problems may precede psychiatric disorders	Hypothesis generation for early intervention and prevention studies in pediatric populations
Schizophrenia → cardiovascular disease profile	Severe mental illness	Heterogeneous	Possible true comorbidity and underdiagnosis	Further investigation of healthcare access, diagnostic pathways, and cardiometabolic monitoring strategies
Schizophrenia → cancer diagnoses	Severe mental illness	Lower recorded incidence	May reflect reduced screening	Evaluation of screening participation and diagnostic equity in severe mental illness
COVID-19 → cognitive disorders	Neuropsychiatric sequelae	Higher incidence	Cognitive effects visible in routine care	Prioritization of prospective follow-up studies and post-infectious care pathways
Depression → cancer or dementia	Psychiatric exposure	Higher incidence	Depression as vulnerability marker	Exploration of shared risk mechanisms and longitudinal prevention strategies
*Ginkgo biloba* → dementia outcomes	Dementia treatment	Slower progression	Observed in longitudinal outpatient data	Hypothesis generation for pragmatic trials and comparative effectiveness research
Antidepressants in bipolar disorder → mania	Bipolar disorder	Variable by drug class	Reflects real-world prescribing heterogeneity	Stratified pharmacoepidemiological and interventional studies
Prodromal features → later dementia	Dementia	Commonly documented	Early signs detectable in primary care	Development of early detection algorithms and risk stratification tools

## Data Availability

No new data were created or analyzed in this study.
